# The optimal diagnostic strategies for patient with coronary artery diseases and stable chest pain syndrome: a cost-effectiveness analysis

**DOI:** 10.1186/s43044-020-00111-y

**Published:** 2020-11-23

**Authors:** Parvin Jafari, Reza Goudarzi, Mohammadreza Amiresmaeili, Hamidreza Rashidinejad

**Affiliations:** 1 Social Determinants of Health Research Center, Institute for Futures Studies in Health, Kerman University of Medical Sciences, Kerman, Iran; 2 Health Services Management Research Center, Institute for Futures Studies in Health, Kerman University of Medical Sciences, Kerman, Iran; 3 Department of Health Management and Economics, Faculty of Management and Medical Informatics, Kerman University of Medical Sciences, Kerman, Iran; 4 Cardiovascular Research center,institute of basic and clinical physiology science., Kerman University of Medical Sciences, Kerman, Iran

**Keywords:** Coronary artery disease, Diagnostic tests, Diagnostic strategies, Cost-effectiveness analysis, Sensitivity analysis

## Abstract

**Background:**

Numerous invasive and noninvasive diagnostic tests with different cost and effectiveness exist for detection of coronary artery disease. This diversity leads to unnecessary utilization of health services. For this reason, this study focused on the cost-effectiveness analysis of diagnostic strategies for coronary artery disease from the perspective of the health care system with 1-year time horizon.

**Results:**

Incremental cost effectiveness ratios of all strategies were less than the threshold except for the electrocardiography-computed tomography angiography-coronary angiography strategy, and cost of the cardiac magnetic resonance imaging-based strategy was higher than the cost of other strategies. Also, the number of correct diagnosis in the electrocardiography-coronary angiography strategy was higher than the other strategies, and its ICER was 15.197 dollars per additional correct diagnosis. Moreover, the sensitivity analysis found that the probability of doing MRI and sensitivity of the exercise electrocardiography had impact on the results.

**Conclusion:**

The most cost-effective strategy for acute patient is ECG-CA strategy, and for chronic patient, the most cost-effective strategies are electrocardiography-single photon emission computed tomography-coronary angiography and electrocardiography-exercise electrocardiography-coronary angiography. Applying these strategies in the same clinical settings may lead to a better utilization of resources.

## Background

In the last decade, cardiovascular diseases have become one of the factors which threaten human health [1].One of the most common cardiovascular diseases is coronary artery disease which occurs due to the accumulation of masses of lipids such as cholesterol and fibrous tissue in the form of plaque on the artery walls and which provides problem for the blood flow in the vessels [2]. Economic burden of coronary artery disease on health care system is significant. For example, in Australia in 2014, coronary artery disease was responsible for 27% of the health care spending [3]. Also, in 2016, a UK study reported £ 62,210 and £ 35,549 cost attributable to coronary artery heart diseases for low- and high-risk patients respectively [4]. An Iranian study in 2017 indicated that cost of this disease was approximately between 4715 and 4908 billion dollars [5].

Every day, a large number of people with chest pain refer to heart centers with near half of them without a real cardiac problem. Hence, correct diagnosis and appropriate treatment of these patients make challenges not only for physicians and hospitals but also for governments, health-insurance companies, and health maintenance organizations [6].

According to available guidelines, diagnostic tests for coronary artery disease include the following: electrocardiography (ECG), echocardiography (ECHO), exercise electrocardiography (Ex-ECG), computed tomography coronary angiography (CTA), coronary angiography (CA), cardiac single-photon emission computed tomography (SPECT), stress cardiac magnetic resonance imaging (C-MRI), exercise echocardiography (EX-ECHO), and stress echocardiography (stress ECHO) [7–9]. These tests with having different cost and effectiveness might lead to unnecessary utilization of health services and impose an enormous economic burden on families, health care systems, and government. For this, optimal allocation of health resources has become important issues for the health care system [10]. Economic evaluation is one of the explicit methods for resource allocation. Economic evaluations are widely employed in health policies, including the evaluation of preventive and diagnostic programs, intervention, treatment, and decision-making. The most commonly used form of economic evaluation is the cost-effectiveness analysis [11]. Because there is no information about cost-effectiveness of the diagnostic tests in Iran, the aim of this study was to evaluate the cost effectiveness of seven diagnostic tests including: ECG, ECHO, Ex-ECG, CTA, CA, SPECT, and C-MRI that are the most common in Iran.

## Methods

This study is a cost-effectiveness analysis from the viewpoint of health care system over a 1-year time horizon.

### Strategies

For the purpose of the present study, nine diagnostic strategies were selected. Relevant data were derived from the medical records (2017–2018) of two Iranian hospitals in 2019. Each of the strategies comprised of two to four diagnostic tests out of seven available tests (ECG, ECHO, EX-ECG, CTA, C-MRI, CA, and SPECT) (see Table 1). All of the strategies started with electrocardiography; however, next steps of the strategy depended on the result of its precedent, i.e., if positive or uncertain result is achieved, strategy will continue. For example, for the patient with chest pain, ECG test is done. If the initial test is positive or uncertain, then ECHO is performed. If ECHO test is also positive, the patient would be subjected to CA.

**Table 1:** Diagnostic test in each strategy

Strategy	First test	Second test	Third test	Fourth test
1	ECG^c^	Echo^b^	CA^a^	
2	ECG	CA		
3	ECG	SPECT^d^	CA	
4	ECG	Echo	SPECT	CA
5	ECG	Echo	EX-ECG^e^	CA
6	ECG	EX-ECG	CA	
7	ECG	EX-ECG	SPECT	CA
8	ECG	CTA^f^	CA	
9	ECG	CMRI^g^	CA	

^a^Invasive coronary angiography

^b^Echocardiography

^c^Electrocardiography

^d^Single-photon emission computed tomography

^e^Exercise electrocardiography

^f^Computed tomography coronary Angiography

^g^Cardiac magnetic resonance imaging

### Modeling

**Fig. 1:**
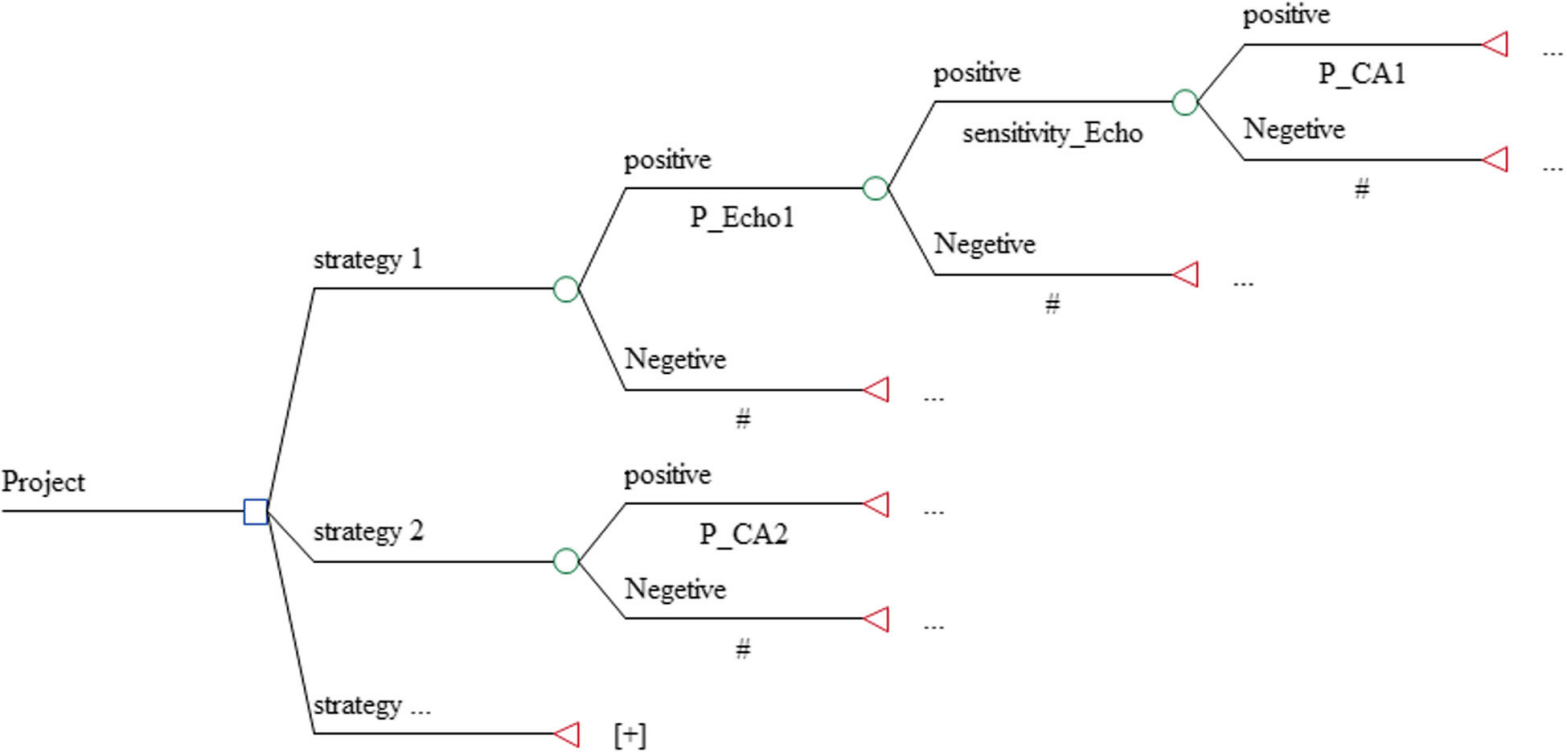
Decision analytic tree for diagnostic strategies: CA (invasive coronary angiography), C-MRI (cardiac magnetic resonance imaging), CTA (computed tomography coronary angiography), ECHO (echocardiography), Ex-ECG (exercise electrocardiography), SPECT (single-photon emission computed tomography), ECG (electrocardiography)

Decision tree was used for modeling, which consisted of nine branches, each one representing a unique strategy. All strategies consisted of several sub-branches, and for each of them, costs, effectiveness, and probabilities were entered into the model. Since all patients underwent electrocardiographic test on arrival at the hospital, it was not included in the modeling, but its cost was calculated for all of the strategies (see Fig. 1).

### Parameters

Model parameters included tests sensitivity, real or false positive probability, cost, and effectiveness. Values of probabilities, costs, and effectiveness were calculated based on available data whereas sensitivities were extracted from previous studies (see Table 2).

**Table 2:** Input data for decision tree model

Parameters	Mean	Min	Max	Distribution	α	β	Source
Probabilities							
ECHO^b^	0.2	0.198	0.199				^a^
SPECT^c^	0.291	0.166	0.418				^a^
EX-ECG^d^	0.146	0.057	0.210				^a^
CTA^e^	0.031	0.031	0.031				^a^
CMRI^f^	0.636	0.636	0.636				^a^
CA^g^	0.98	0.96	1.000				^a^
Sensitivity, %							
EX-ECG	65	42	92				[12]
ECHO	50	32	68				[13]
CTA	88	83	92				[12]
SPECT	87	48	88				[12]
CMRI	89	88	94				[12]
CA	100	100	100				[12]
Cost ($)							
ECG	0.793	0.026	7.401	Gama			
EX-ECG	17.480	10.045	41.398	Gama			^a^
ECHO	33.090	8.170	220.609	Gama			^a^
CTA	186.610	185.203	188.018	Gama			^a^
SPECT	217.845	20.045	282.492	Gama			^a^
C-MRI	54.179	53.572	55.780	Gama			^a^
CA	187.686	148.034	243.199	Gama			^a^
Effect, %							
Strategy 1	19.51			Beta	0.195	0.496	^a^
Strategy 2	96.903			Beta	0.195	0.496	^a^
Strategy 3	40.862			Beta	0.195	0.496	^a^
Strategy 4	3.187			Beta	0.195	0.496	^a^
Strategy 5	1.065			Beta	0.195	0.496	^a^
Strategy 6	20.388			Beta	0.195	0.496	^a^
Strategy 7	5.072			Beta	0.195	0.496	^a^
Strategy 8	2.994			Beta	0.195	0.496	^a^
Strategy 9	63.636			Beta	0.195	0.496	^a^

^a^Calculated in this study (study’s data)

^b^Echocardiography

^c^Single-photon emission computed tomography

^d^Exercise electrocardiography

^e^Computed tomography coronary Angiography

^f^Cardiac magnetic resonance imaging

^g^Invasive coronary angiography

#### Costs

All costs associated with inpatient and outpatient services were considered from the perspective of the health care system. These included direct medical and non-medical costs.

Direct medical costs encompassed cost of labor, laboratory, pathology, pharmaceuticals, medical goods and equipment, hospitalization, and diagnostic imaging. Direct non-medical costs included cost of capital depreciation, energy consumption, and administrative affairs*.*

Direct medical costs were collected by referring to the medical records department using the patient records, and direct non-medical costs were collected from the hospital accounting department. Finally, these costs were calculated for each method and strategy separately.

#### Effectiveness

Effectiveness was measured by the number of cases who were correctly diagnosed because angiography is considered a gold standard with 100% sensitivity [14], and given that all strategies ultimately end to angiography, if the angiographic result was positive, it showed that the person had been correctly diagnosed, and if it was negative, indicated that the patient had not been correctly diagnosed.

### Cost-effectiveness analysis

Incremental cost effectiveness ratio (ICER) was calculated with the following formula [15]:

$$ \mathrm{ICER}=\frac{Cn- Cc}{En- Ec} $$ in which:

Cn = cost of new intervention

En = effect of new intervention

Cc = cost of current

Ec = effect of current

According to previous studies [12, 14, 16], clinical guidelines, and expert opinions, we found out that strategies 1 and 2 can be used to diagnose cases with acute coronary syndrome and for diagnosing case with chronic coronary syndrome; strategies 3–9 are helpful; hence, diagnostic strategies were analyzed in three categories of total, acute, and chronic.

An expert panel made up of cardiologists, economists, and policymakers suggested the cost of sixth strategy as the baseline with its maximum cost to be regarded as threshold. Therefore, the threshold of the study was set at 2600 $ per correct diagnosis. All data analysis was performed using decision tree model through TreeAge pro 2011.

### Sensitivity analysis

**Fig. 2:**
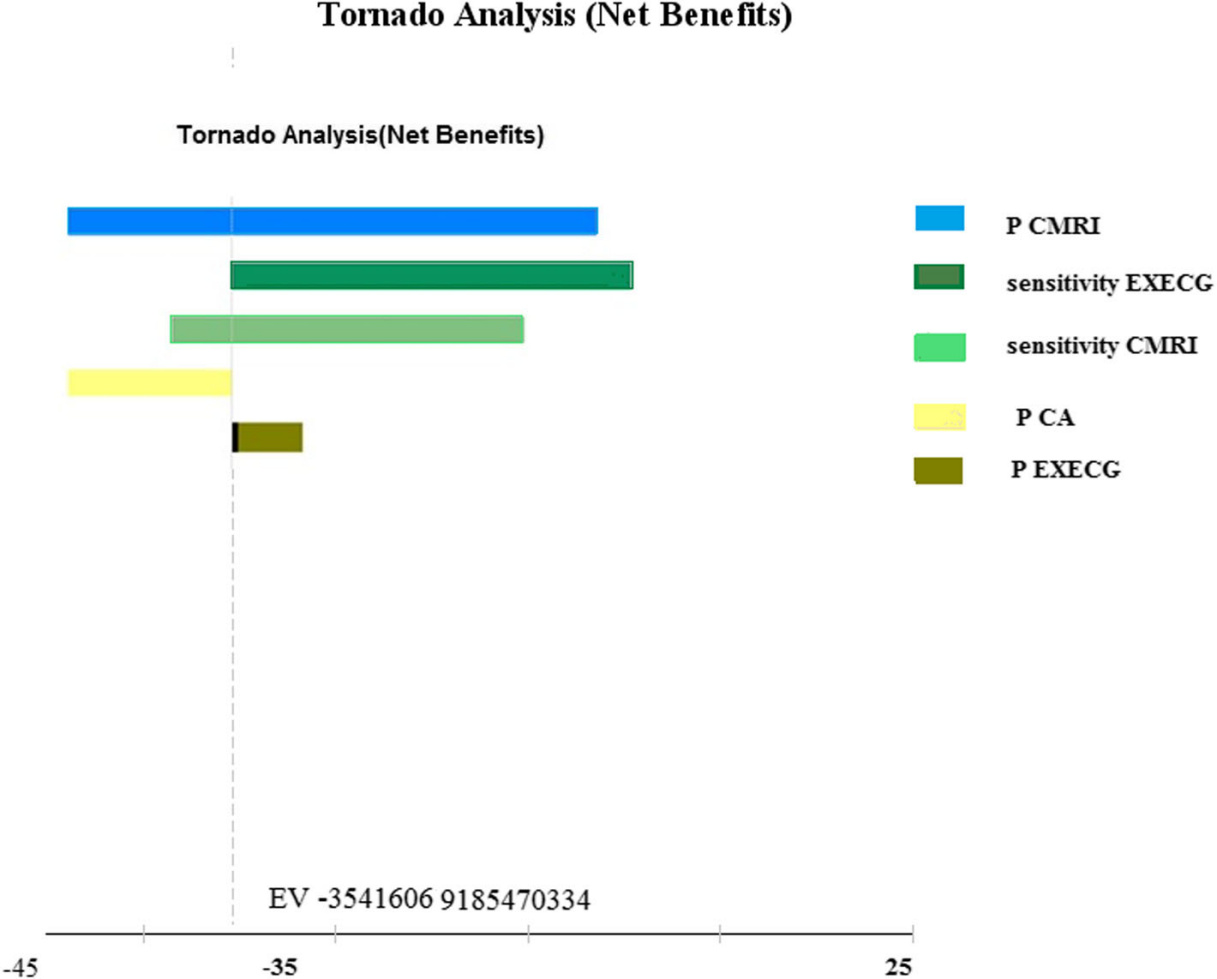
Tornado diagram: P (probability), CA (invasive coronary angiography), C-MRI (cardiac magnetic resonance imaging), CTA (computed tomography coronary angiography), ECHO (echocardiography), Ex-ECG (exercise electrocardiography), SPECT (single-photon emission computed tomography), ECG (electrocardiography)

To test the robustness of the model, the impact of uncertain parameters such as cost, effectiveness, sensitivity, and probabilities on results were assessed. The parameters were analyzed by tornado diagram (Fig. 2); finally, considering the output of the tornado diagram, parameters that had the most effect on the model were analyzed by one-way and two-way sensitivity analysis. Also, probability sensitivity analysis with Monte Carlo simulation was performed for cost parameters using gamma distribution and effectiveness parameters using beta distribution.

## Results

### Base case results

Analysis indicated that ECG–Echo–EXECG–CA strategy with $48.183 cost and 0.003% correct diagnosis had the minimum cost and effectiveness, so it was chosen as the current strategy.

ECG-CA strategy with 93.899% correct diagnoses is the most effective strategy. ECG-CMRI-CA strategy had the highest cost. ICER of ECG-CTA-CA strategy vs strategy 5 is 94.450 dollars per additional case that is above the threshold and is not acceptable. ICER of ECG-CA strategy is 15.197 dollars per case, and it is the most cost-effective strategy.

The other information is available in Table 3.

**Table 3:** Base case cost-effectiveness analysis

	Cost ($)	Incremental cost	Effectiveness	Incremental effectiveness	ICER	ACER
Total strategies						
Strategy 5	48.183	-	0.003	-	-	16,061.091
Strategy 4	104.076	55.893	0.043	0.04	1397.342	2420.394
Strategy 7	108.378	60.195	0.137	0.342	176.009	791.084
Strategy 6	166.415	118.232	2.572	2.568	46.040	64.702
Strategy 8	255.088	206.905	0.078	0.074	2796.019	3270.367
Strategy 1	364.571	316.388	1.896	1.893	167.135	192.284
Strategy 3	561.280	513.097	14.488	14.484	35.425	38.741
Strategy 2	1475.128	1426.945	93.899	93.895	15.197	15.709
Strategy 9	3450.017	3401.834	36.020	36.017	94.450	95.780
Acute patient strategies						
Strategy 2	364.571	-	93.899	-	-	3.882
Strategy 1	1475.128	− 1110.557	1.896	-92.002	12.071	778.021
Chronic patient strategies						
Strategy 5	48.18327345	-	0.003	-	-	16,061.09115
Strategy 4	104.0769569	55.89368341	0.043	0.04	1397.342085	2420.394346
Strategy 7	108.3785836	60.19531012	0.137	0.342	176.0096787	791.0845516
Strategy 6	166.4153344	118.232061	2.572	2.568	46.04052219	64.70269613
Strategy 3	561.2803499	513.0970764	14.488	14.484	35.42509503	38.74105121

All strategies were located at the northeast of cost effectiveness acceptability plane. The eighth (ECG-TA-CA) strategy has higher cost, and it is placed above of threshold line and therefore is dominated.

### Sensitivity results

**Fig. 3:**
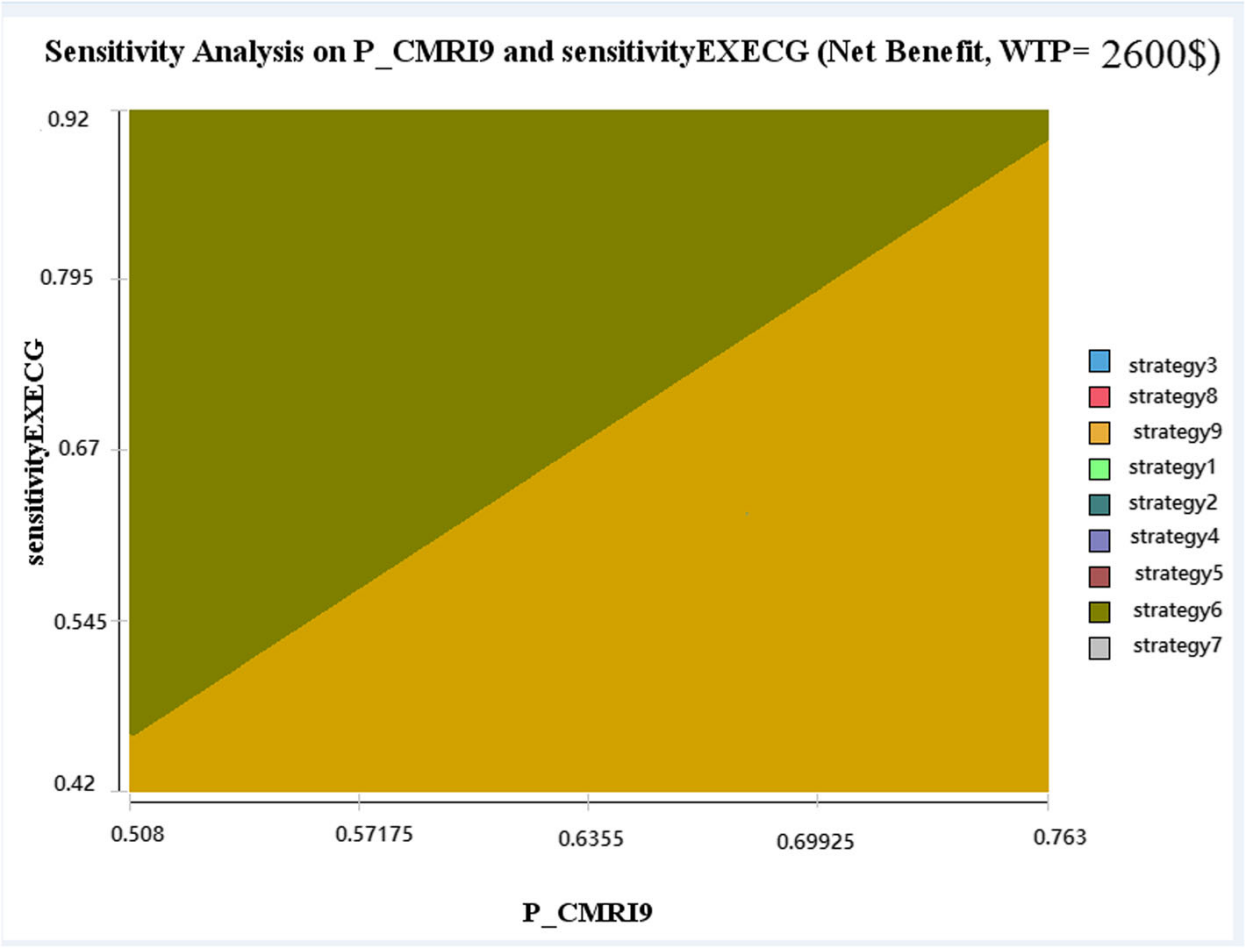
Two-way sensitivity analysis: two-way sensitivity analysis between sensitivity EX-ECG, P, and CMRI

All model parameters were considered in Tornado analysis, but the diagram covered only variables which affected results. According to the diagram, probability of C-MRI, CA, and EX-ECG, and sensitivity of C-MRI and EX-ECG have the most impact on the results of model. One-way sensitivity analysis indicated that probability of MRI and sensitivity of EXECG influenced the ICERs; however, two-way sensitivity analysis showed that by increasing the probability of MRI, the ninth strategy would become more cost effective, and by increasing the sensitivity of EXECG, the sixth strategy would become more cost effective (Fig. 3).

**Fig. 4:**
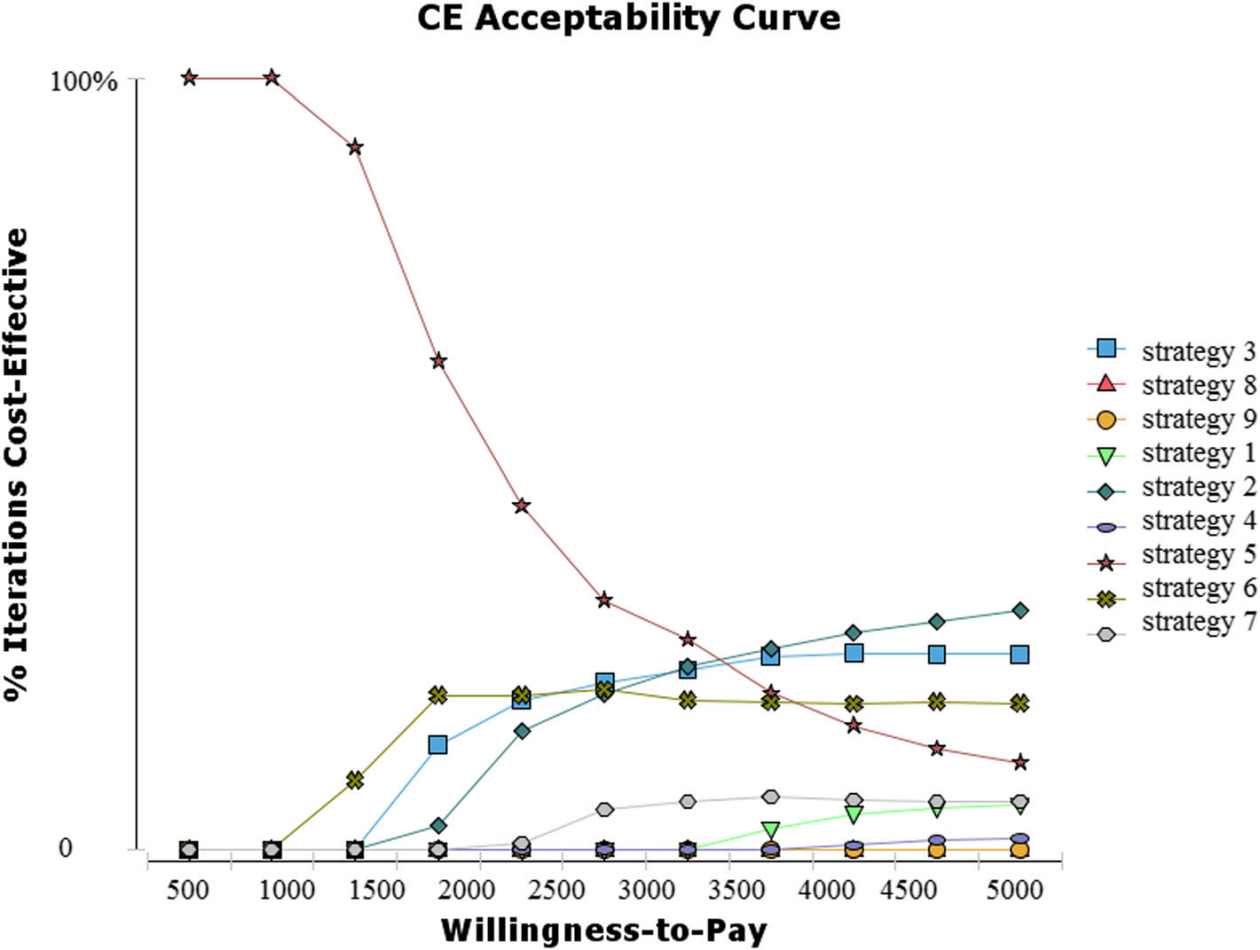
Cost-effectiveness acceptability curve

The cost-effectiveness acceptability curve (Fig. 4) with 1000 iterations showed that the cost-effectiveness probability of strategy 5 (ECG-ECHO-EXECG-CA) under 500$ threshold is 100%. Whereas for the willingness-to-pay higher than the threshold, the cost-effectiveness probability of strategy 2 (ECG-CA), strategy 3 (ECG-SPECT-CA), and strategy 5 is the highest.

## Discussion

This study was done in two Iranian hospitals during 2017–2018, to evaluate and compare the cost and effectiveness of nine diagnostic strategies for coronary artery disease with chest pain. Based on the results, the SPECT test had the highest cost per case, followed by CTA, MRI, ECHO, EX-ECG, and ECG, respectively. Similarly, Boldt et al. [17] and Min et al. [16] showed that the highest cost belongs to SPECT. Zacharias et al. (2015) and Zacharias et al. (2016) indicated that the cost of ECHO was less than the cost of EXECG which contradicts the present study [18, 19].

The MRI-based strategy with the cost of $3450.017 was more expensive than the others followed by the SPECT-based strategy and CTA-based strategy respectively. Likewise, Min et al. showed that CTA-based strategy was costlier than EXECG-based strategy. Bertoldi et al.’s study indicated that cardiac MRI-based strategies and SPECT-based strategies had the highest costs and can be used depending on threshold; the study by Walker et al. showed that the cost of the ninth strategy (MRI), which was $ 18,284, is greater than the cost of the SPECT-based strategy [12, 14, 20]. Moschetti et al., Thom et al., and Boldt et al. also depicted that C-MRI is costly but can be used as a good option to diagnose people with a high probability of coronary artery disease and to reduce angiography [17, 21, 22]. In contrast, Min et al. [20] showed that ECG-CA strategy, at $ 14,003, is more expensive than other strategies, and the study by Genders et al. [23] indicated ECG-CTA-CA strategy was less expensive.

According to the results, it is clear that the cost of tests and diagnostic strategies in Iran is lower than the costs in other countries because of the different types of medical insurances that the government provided for people, and the other reason is because of the majority of hospital staffs have low salary.

In this study, the effectiveness of the second strategy (ECG-CA) was 93.899%, which is higher than the other strategies, followed by MRI-based strategy and SPECT-based strategy. Similarly, Thom et al. showed that rate of correct diagnosis by MRI and ECHO was 80% and 75% respectively [22]. Hamilton et al. showed the rate of correct diagnosis by CTA and EXECG were 26% and 51% respectively [24]. Similar to the findings of current study, Sharples et al. also indicated that the SPECT-based strategy had 83% correct diagnosis [25].

To sum up, for acute patient’s strategies, the cost and effectiveness of the second strategy (ECG-CA) were significantly higher than ECG-ECH-CA strategy. ICERs indicate that the first strategy generates $12.071 more cost per correct diagnosis. Therefore, the second strategy is more cost-effective for patients with acute coronary artery disease.

However, the finding of cost-effectiveness analysis of chronic patients’ strategies suggested that the ECG-ECHO-SPECT-CA and ECG-EX ECG-SPECT-CA strategies were not cost effective and therefore not acceptable because of their higher ICER and ACER. Strategies 3 (ECG-SPECT-CA) and 6 (ECG-EXECG-CA) had an appropriate ICER. Based on the results, it can be concluded that strategies 3 and 6 are more appropriate for people with lower risk and emerged as the most effective strategy.

The American Heart Association’s latest guidelines for diagnosis and management of angina made more traditional recommendations for EXECG as the best first line compared to ECHO and SPECT or CTA [12].

In this study, ECG-CTA-CA strategy had high cost. Because in Iran the threshold for a correct diagnosis is low, this strategy is not cost-effective. Although Min et al. study’s indicated that the eighth strategy even with ICER $ 17 516 per patient is most cost effective, and also, the studies of Priest et al., Joseph et al., Hamilton et al., and Min et al. showed that the ECG-CTA-CA strategy is the most cost-effective strategy [16, 20, 24, 26, 27].

A possible limitation of this study was that we did not include all diagnostic strategies and only analyzed strategies which are more common in Iran. Another limitation of the present study is the fact that test sensitivity was extracted from other studies which may be different from the real one. This study was carried out from economic aspect so it might have different results according to the patient’s situation. Given that the study did not assess the clinical outcomes, three important concepts are lacking such as the quality-adjusted life years (QALY) assessment, standard gambling (SG), and time-trade off (TTO) concept.

Despite these limitations, to our knowledge, this study is the first study to evaluate the cost-effectiveness of coronary artery disease diagnostic tests in Iran and also this research has analyzed the diagnostic strategies of coronary artery disease in general as well as in two groups of acute and chronic patient strategies.

## Conclusion

This study indicated that all strategies except CTA-based strategy are cost-effective, but the ECG-CA strategy is the most cost-effective strategy for acute patients. For chronic patients, ECG-SPECT-CA and ECG-EX ECG-CA strategies are the best choices. Due to the limited resources in the health care system, applying these strategies to patient in the same clinical setting may lead to a better utilization of resources. Strategy 9 (ECG-CMRI-CA) in high threshold may lead to early diagnosis of the disease and thereby saving resource. To summarize, it is recommended to consider economic issues as well a clinical issues for choosing diagnostic strategies, and in the same condition, the cost effectiveness of the strategies should be the basis of choice. Due to the difference in cost effectiveness of diagnostic strategies in Iran compared with other countries, these results should be included in developing local clinical guidelines.

## Data Availability

The datasets used and/or analyzed during the current study are available from the corresponding author on reasonable request.

## Abbreviations

ICER: Incremental cost-effectiveness ratio
ACER: Average cost-effectiveness ratio
ECG: Electrocardiography
EX-ECG: Exercise electrocardiography
SPECT: Single-photon emission computed tomography
ECHO: Echocardiography
CTA: Computed tomography coronary angiography
CMRI: Cardiac magnetic resonance imaging
CA: Invasive coronary angiography
P: Probability
QALY: Quality-adjusted life years
SG: Standard gambling
TTO: Time-trade off
